# Subjective outcome after laparoscopic hiatal hernia repair for intrathoracic stomach

**DOI:** 10.1007/s00423-016-1504-2

**Published:** 2016-11-09

**Authors:** P. S. S. Castelijns, J. E. H. Ponten, M. C. G. Van de Poll, S. W. Nienhuijs, J. F. Smulders

**Affiliations:** 1 Departement of Surgery, Catharina Hospital Eindhoven, Michelangelolaan 2, 5623 EJ Eindhoven, The Netherlands; 2 Departement of Surgery, MUMC+, P. Debyelaan 25, 6229 HX Maastricht, The Netherlands; 3 Department of Intensive Care Medicine, MUMC+, P. Debeyelaan 25, 6229 HX Maastricht, The Netherlands; 4 School of Nutrition and Translational Research in Medicine, Maastricht University, Maastricht, The Netherlands

**Keywords:** Intrathoracic stomach, Hiatal hernia, Nissen, Fundoplication, Quality of life, Long-term follow-up

## Abstract

**Purpose:**

For decades, an intrathoracic stomach (ITS) has been a definite indication for surgery due to the perceived risk of an acute volvulus with perforation, gangrene, or hemorrhage. At the present time, elective laparoscopic repair is the first choice for treatment of ITS. There is a lack of evidence in the long-term quality of life after a hiatal hernia repair for an intrathoracic stomach.

**Methods:**

A retrospective analysis was performed on all patients undergoing a hiatal hernia repair for an intrathoracic stomach between January 2004 and January 2015. Additionally, to a hiatal closure, the patients received an antireflux procedure. Outcome measures included patient characteristics, operative details, complications, and postoperative morbidity and mortality. All patients were sent a quality of life questionnaire to assess long-term quality of life and patient satisfaction. A higher quality of life score represents a better quality of life.

**Results:**

Eighty-six patients underwent laparoscopic repair for ITS, from which, one patient died during surgery. Eighty-five patients were contacted and 81 completed the questionnaire, resulting in a response rate of 95.3 %. At a median follow-up of 2.7 years (range 0.1–9.6), the mean quality of life score was 13.5 (standard deviation 2.8). The mean overall satisfaction was 8.4.

There were four recurrences: three in the first 12 days after surgery and one in 2.4 years.

**Conclusions:**

Very good results in patient satisfaction and symptom reduction were achieved after a median follow-up of 2.7 years in this laparoscopic repair of the intrathoracic stomach single center experience study. The symptomatic recurrence rate was very low.

## Introduction

For decades, an intrathoracic stomach (ITS) has been a definite indication for surgery due to the perceived risk of an acute volvulus with perforation, gangrene, or hemorrhage [1, 2]. Due to the increasing incidence of gastro esophageal reflux disease (GERD), more people undergo diagnostic workups for reflux symptoms. One of the causes for the increasing incidence of GERD is the exponentially growing problem of obesity. In some of these patients, the GERD symptoms are caused by the large hiatal hernia rather than by obesity itself. An intrathoracic stomach may also be found as an accidental finding. These developments resulted in an increased number of patients with an intrathoracic stomach [3].

The increasing availability of minimal invasive surgery has lowered the threshold to perform surgery on these patients. However, whether these patients with minimal symptoms actually benefit from such surgical treatment of intrathoracic stomach is unclear.

The first laparoscopic paraesophageal hernia (PEH) repair was reported in 1992 by Cuschieri et al. [4]. Over the succeeding years, antireflux surgery with repair of the hiatal hernia has become the standard treatment for all types of hiatal hernias [5]. The symptomatic outcome of antireflux surgery for GERD is well documented in a large series of cases and clinical trials [6]. Nevertheless, only data regarding the recurrence rates for an intrathoracic stomach repair are available. There is a lack of substantial data describing the quality of life after elective surgery for intrathoracic stomach. The largest current series describes a cohort of 73 patients with a reported objective follow-up of 5 years [7]. However, only 33 patients completed the 3-year follow-up, and only 12 patients completed the entire 5-year follow-up.

In this article, we reported on a large series of patients with intrathoracic stomachs who underwent an elective minimal invasive surgery. We presented a long-term follow-up data with a special emphasis on symptomatic outcome and patient satisfaction. These factors were assessed by a standardized questionnaire.

## Materials and methods

We included patients who underwent surgery for a primary intrathoracic stomach, defined as >50 % of the stomach into the thorax on barium swallow investigation or on CT scan [5]. All the data was retrieved from the hospital information system at our institution for patients who were operated on between January 1, 2004 and January 1, 2015. Parameters that were extracted included patient characteristics, preoperative symptoms, preoperative medication usage, and diagnostic workups.

Patients received a standardized questionnaire by mail. This questionnaire was a modified version of the one that was used by Mittal et al. [7] (Appendix 1). Items on the questionnaire included heartburn, regurgitation, dysphagia, retrosternal pain, gas bloating, and the use of antiacids. In addition to these questions, patients were requested to score the result of the procedure on a visual analog scale (VAS). Patients who did not respond were contacted by phone up to three times to maximize the response rate.

### Surgical technique

All procedures were performed laparoscopically. After repositioning the stomach and dissecting the hernia sac, the hiatus was closed using non-absorbable woven sutures (Ti-cron™, Covidien, New Haven, CT, USA). In several cases, the cruroplasty was reinforced using a prosthetic mesh (Parietex™, Covidien) at the discretion of the operating surgeon. This was then followed by a 360-degree floppy Nissen fundoplication. We did not perform gastropexy or a gastrostomy. The most distal suture fixates the wrap to the wall of the esophagus to prevent telescoping.

### Immediate postoperative period

Patients were put on a liquid diet for 2 weeks. Operation time, complications during surgery, length of stay (LOS), in-hospital postoperative complications, and readmissions were retrieved from the hospital information system.

### Statistics

A retrospective database was managed in Access 2010 (Microsoft, USA). All data was analyzed using SPSS for MacOs version 21.0 (SPSS Inc., Chicago, IL, USA).

## Results

### Patient characteristics

A total number of 86 patients were operated on. One patient died during the procedure. Out of the 85 patients that were contacted (20 were male), 81 (18 were male) responded, concluding a 95.3 % response rate. Data are reported on respondents only.

The median age was 63 years (42–80). The median BMI was 27 (20–42). Twenty patients (24.7 %) received a mesh-based repair. The two most dominant symptoms prior to surgery were retrosternal pain and dysphagia. Baseline characteristics are summarized in Table 1.

**Table 1:** Baseline characteristics

	Overall (*n* = 81)	Mesh (*n* = 20)	Suture (*n* = 61)	*P* value
Male/female ratio	18/63	5/15	13/48	0.761^a^
median age	63 (42–80)	65 (43–80)	62.5 (42–77)	0.273^b^
median BMI	27 (20–42)	28.5 (22–42)	27.0 (20–41)	0.343^b^
Median ASA classification (range)	2 (1–3)	2 (2–3)	2 (1–3)	0.033^a^
Diabetes	5/81 (6.2 %)	1/20 (5 %)	4/61 (6.6 %)	1.000^a^
Smoking	7/81 (8.6 %)	2/20 (10 %)	5/61(8.2 %)	1.000^a^
Asthma/COPD	16/81 (19.8 %)	4/20 (20 %)	12/61 (19.7 %)	1.000^a^
Heartburn	24/81 (29.6 %)	6/20 (30 %)	18/61 (29.5 %)	0.967^c^
Regurgitation	12/81 (14.8 %)	3/20 (15 %)	9/61 (14.8 %)	1.000^a^
Dysphagia	29/81 (35.8 %)	7/20 (35 %)	22/61 (36.1 %)	0.931^c^
Retrosternal pain	29/81 (35.8 %)	10/20 (50 %)	19/61 (31.1 %)	0.127^c^
Epigastric pain	23/81 (28.4 %)	4/20 (20 %)	19/61 (31.1 %)	0.337^a^
Nausea/vomiting	24/81 (29.6 %)	6/20 (30 %)	18/61 (29.5 %)	0.967^c^
PPI Usage	58/81 (71.6 %)	16/20 (80 %)	42/61 (68.9 %)	0.337^c^

*BMI* body mass index, *ASA* American Society of Anesthesiologists, *COPD* chronic obstructive pulmonary disease, *PPI* proton pump inhibitor

^a^Fisher exact

^b^Mann-Whitney

^c^Chi-squared

### Quality of life

**Fig. 1:**
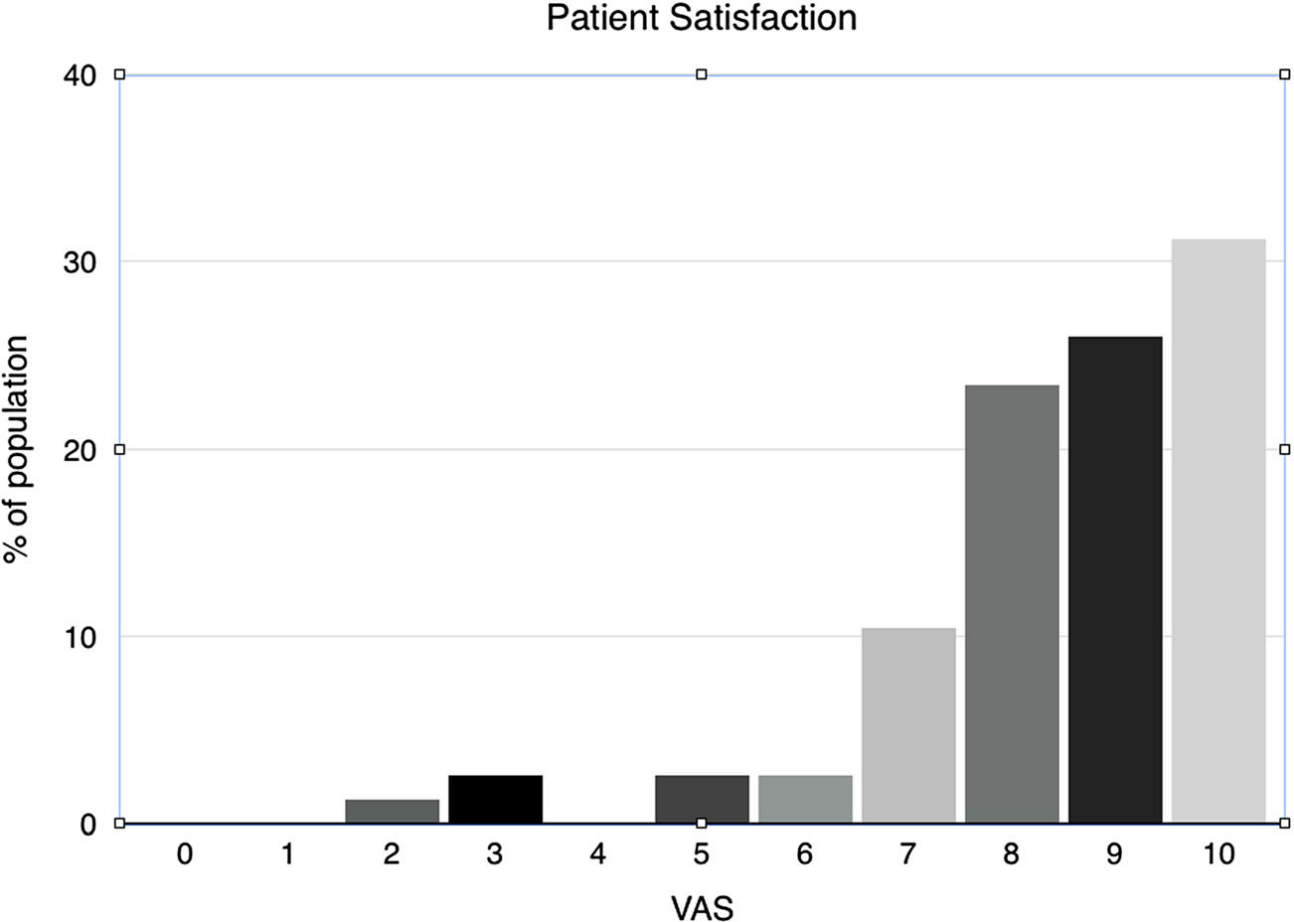
Patient satisfaction score

**Fig. 2:**
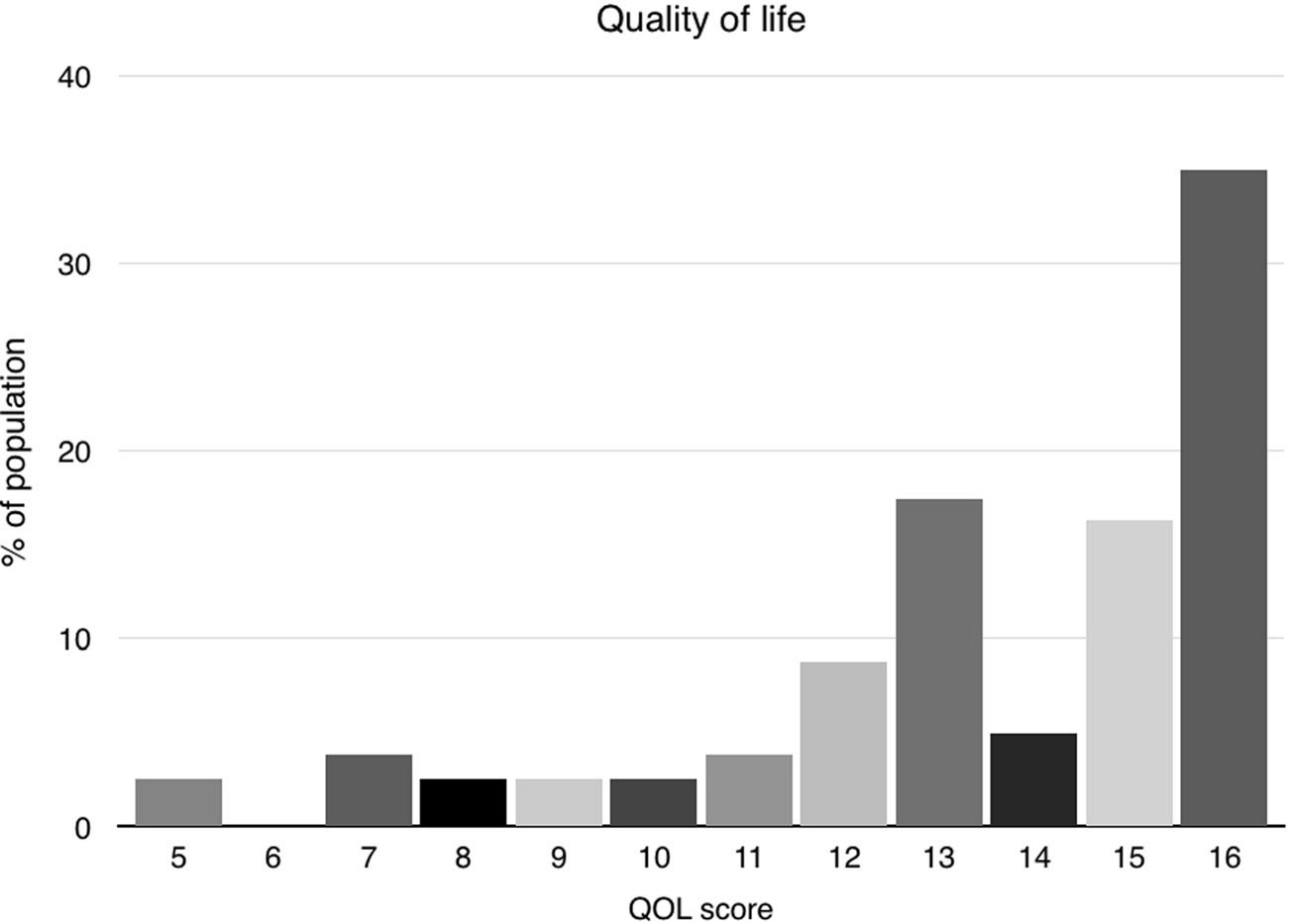
Quality of life score

After a median follow-up of 2.7 years ranging from 48 days to 9.6 years, overall satisfaction assessed by the VAS was 8.4 (Fig. 1). Most patients experienced minor to no symptoms, providing a mean quality of life (QoL) score of 13.5 (SD 2.8). A score of 16 indicates maximum quality of life and no complaints (Fig. Fig. 2). The mean QoL assessment score was 13.5 for mesh reinforced cruroplasty and 13.7 for non-mesh reinforced cruroplasty. (*p* = 0.875) The satisfaction on the VAS was 8.2 and 8.5, respectively. No statistically significant difference in QoL was found between both groups. Since one patient died during surgery, follow-up data of the remaining 80 patients are presented. All details regarding the quality of life are demonstrated in Table 2. In the analysis of the short-term results, there were no statistically significant differences between both groups (Table 3). We did not find a difference in quality of life between the patients with short-term follow-up compared to the long-term results (Table 4). Since there were no patients with a mesh reinforcement in the long-term group, we compared this group with the patients that did not receive a mesh reinforcement as well in the short-term group. This in order to perform a more adequate analysis.

**Table 2:** Symptoms and QoL at follow-up—Mesh vs non-mesh

Symptom	Mean symptom score *n* = 80 (SD)	Mesh *n* = 19 mean (SD)	Suture *n* = 61 mean (SD)	*P* value
Dysphagia	2.7 (0.7)	2.6 (0.8)	2.7 (0.7)	0.814^a^
Heartburn	2.5 (0.9)	2.5 (1.0)	2.5 (0.9)	0.599^a^
Regurgitation	2.6 (0.8)	2.7 (0.8)	2.5 (0.8)	0.247^a^
Retrosternal pain	2,6 (0.6)	2.5 (0.6)	2.7 (0.6)	0.466^a^
Nausea/vomiting	2.5 (0.8)	2.6 (0.7)	2.5 (0.8)	0.911^a^
Gas bloating	26/80 (32.5 %)	8/19 (42.1 %)	18/61 (29.5 %)	0.306^c^
Overall satisfaction	8.4 (1.7)	8.2 (1.6)	8.5 (1.7)	0.253^b^
Total QoL score	13.5 (2.8)	13.5 (3.0)	13.7 (2.8)	0.875^b^
PPI usage	33/79 (41.8 %)	8/19 (42.1 %)	25/60 (41.7 %)	0.973^c^

*QoL* quality of life, *SD* standard deviation, *PPI* proton pump inhibitor

^a^Fisher exact

^b^Mann-Whitney

^c^Chi-squared

**Table 3:** Symptoms and QoL at follow-up <5 years

Symptom	Mean symptom score *n* = 66 (SD)	Mesh *n* = 19 mean (SD)	Suture *n* = 47 mean (SD)	*P* value
Dysphagia	2.6 (0.7)	2.6 (0.8)	2.6 (0.7)	0.902^a^
Heartburn	2.5 (0.9)	2.5 (1.0)	2.5 (0.8)	0.504^a^
Regurgitation	2.6 (0.8)	2.7 (0.8)	2.6 (0.7)	0.550^a^
Retrosternal pain	2.6 (0.6)	2.5 (0.6)	2.7 (0.6)	0.386^a^
Nausea/vomiting	2.5 (0.8)	2.6 (0.7)	2.5 (0.8)	1.000^a^
Gas bloating	22/66 (33.3 %)	8/19 (42.1 %)	14/47 (29.8 %)	0.336^b^
Overall satisfaction	8.4 (1.8)	8.2 (1.6)	8.5 (1.9)	0.539^d^
Total QoL score (mean/range)	13.6 (2.9)	13.5 (3.0)	13.6 (2.9)	0.660^c^
PPI usage	24/65 (36.9 %)	8/19 (42.1 %)	16/46 (34.8 %)	0.578^b^

*QoL* quality of life, *SD* standard deviation, *PPI* proton pump inhibitor

^a^Fisher exact

^b^Chi-squared

^c^Mann-Whitney

^d^Independent sample *t* test

**Table 4:** Symptoms and QoL <5 years vs >5 years for suture repair only

Symptom	>5 years *n* = 14 mean (SD)	<5 years *n* = 47 mean (SD)	*P* value
Suture/mesh	14:0	47:0	
Follow-up	7.0 (1.7)	2.6 (1.4)	<0.05^a^
Dysphagia	2.8 (0.6)	2.6 (0.7)	1.000^b^
Heartburn	2.4 (1.0)	2.5 (0.8)	0.761^b^
Regurgitation	2.3 (0.8)	2.6 (0.7)	0.275^b^
Retrosternal pain	2.7 (0.5)	2.7 (0.6)	0.756^b^
Nausea/vomiting	2.4 (0.6)	2.5 (0.8)	0.141^b^
Gas bloating	4/14 (28.6 %)	14/47 (29.8 %)	1.000^b^
Overall satisfaction (median/range)	8.7(1.1)	8.5(1.9)	0.827^c^
Total QoL score (mean/range)	13.3 (2.4)	13.6 (2.9)	0.317^c^
PPI usage	9/14 (64.3 %)	16/46 (34.8 %)	0.050^d^

*QoL* (quality of life), *SD* (standard deviation), *PPI* (proton pump inhibitor)

^a^Independent sample *t* test

^b^Fisher exact

^c^Mann-Whitney

^d^Chi-squared

### Operative outcome

All procedures were performed in a non-emergency setting by the senior author (JS) or under his supervision. The median operative time was 89 min (range 53–212 min). Twenty patients received a mesh-reinforced cruroplasty, whereas 61 patients received a pure suture repair of the hiatal hernia. The decision to use a mesh was made during the surgery at the discretion of the operating surgeon. The reason for using a mesh reinforcement was weakness of the right crus that was noticed in six patients and a very large defect (>8 cm) in 14 patients. Nine minor complications and two major complications were reported, resulting in a complication rate of 12.3 %. Minor complications included a serosal injury and opening of the pleura, while major complications included a laceration of the esophagus and a perforation of the aorta. The laceration of the esophagus was treated by an endoluminal stent. The perforation of the aorta occurred during the mobilization of the esophagus, where there were adhesions between the aorta and the esophagus. A conversion to open surgery was performed; however, the damage to the aorta was too extensive and the patient died during surgery. Autopsy revealed an aneurysm, which was unknown prior to surgery.

Operative details are summarized in Table 5.

**Table 5:** Operative details (*n* = 82)

	No. of patients
Surgery type	
Open	0
Laparoscopic	82
Conversion	1
Elective	82
Emergency	0
Antireflux surgery	
Nissen	81
Hiatal closure	
Suture	61
Mesh based	20
Number of sutures (median/range)	
Suture	3 (2–6)
Mesh reinforcement	4 (0–6)
Median operative time (range)	
Suture	86.5 (53–212)
Mesh	95.0 (68–126)
Complications	
Opening pleura	6
Serosal damage	2
Bleeding	2
Laceration of the esophagus	1
Mortality	1

### Postoperative period

Postoperative complications were reported in 12.5 % of the patients. Most reported postoperative complaints were nausea and chest pain, but these were not classified as complications. Five pneumothoraces occurred of which, two were treated by chest tube drainage and three resolved with supportive therapy. The one patient that suffered from an intraoperative esophageal perforation developed a sepsis due to persistent leakage and, subsequently, underwent an esophageal resection with a gastro-thoracic reconstruction. Three recurrences were seen within 12 days, and all were reoperated on. Two of them were within the same admission. The median hospital stay was 2days (range 1–48 days).

### Mortality

One patient died during the procedure due to perforation of the aorta. Emergency laparotomy was performed, but the patient could not be saved.

### Reoperation

Apart from the three early recurrences mentioned above, five other patients underwent a reoperation during their follow-up. The total percentage of reoperation was therefore 9.9 %. Two patients were operated on because of persistent dysphagia. Two other patients were operated on for GERD due to failure of the fundoplication, while the last one underwent a reoperation for a late recurrent hiatal hernia (868 days after the initial procedure). No symptomatic recurrences were seen after the mesh-reinforced cruroplasty. All causes for reoperation along with their corresponding follow-ups are presented in Table 6. Median quality of life after reoperation was 12 compared to a median of 15 in patients that did not undergo reoperation (*p* = 0.098).

**Table 6:** Reoperations

Causes of reoperation	Follow-up (days)
Failure wrap, crus intact	252
Torsion wrap, crus intact	1164
Early recurrence, due to vomit	2
Wrap in thorax	5
Rupture crus suture	868
Persistent dysphagia, redo Toupet, remove one suture from crus	439
Persistent dysphagia, redo Toupet	238
Wrap in thorax, failure crus sutures	12

## Discussion

In this paper, we report one of the largest single center experiences on laparoscopic repair of an intrathoracic stomach accompanied by a great response rate. We found very good results in patient satisfaction and quality of life after a median follow-up of 2.7 years.

Nissen fundoplication in patients with GERD is proven effective and leads to a great symptomatic outcome [8]. The quality of life in patients with GERD is measured by the HRQL-GERD questionnaire, which is a validated list of questions regarding the most common symptoms. However, since patients with ITS reported different symptoms at baseline, this questionnaire is not suitable in our population. There is still no validated questionnaire available that represents the quality of life in patients with an intrathoracic stomach. Therefore, we used a modified standardized questionnaire that was previously published by Mittal et al. [7] (Appendix 1). In this questionnaire, the most common symptoms in patients with an intrathoracic stomach were scored. The quality of life we found in our large cohort of patients was in line with the great results described previously [7].

Due to the retrospective aspect of this study, we did not have the preoperative quality of life assessment. For this reason, we were unable to compare our postoperative results with the preoperative data that we retrieved from the patient information system. This is one of the most limiting aspects of this study. Nonetheless, if we compared our results with the data reported by Mittal et al., who used the same questionnaire, we would see comparable results. They reported a mean satisfaction score of 9.0 at a 3-year follow-up compared to a mean satisfaction of 8.4 for our cohort. The individual symptom scores were also comparable at the 3-year follow-up [7].

Three patients had an early recurrence in our study. This is probably due to persistent postoperative vomiting, which increases the intra-abdominal pressure, leading to reherniation of the wrap into the thorax. Iqbal et al. has described a relation between failure of the procedure and postoperative nausea and vomiting (PONV) prevention after a Nissen fundoplication in 2006 [9]. They concluded that it is important to prescribe antiemetics after the procedure. Antoniou et al. described that postoperative vomiting, as a result of the procedure, only occurred on the first postoperative day [10]. However, we believe that antiemetics might need to be prescribed for a longer period of time, since we have seen early recurrences due to vomiting up to 12 days.

We noticed only one late recurrence after a follow-up of 2.4 years. The total recurrence rate is therefore 4.9 % and is comparable with the reported median recurrence rate of 7 % (range 0–42 %) reported by Draaisma et al. in a large review describing 32 studies [11]. Not all of our patients received a standard objective follow-up by means of radiology investigations. We therefore may have underestimated the anatomical recurrence rate, especially since several studies demonstrated a very poor relationship between symptoms and the presence of an anatomical recurrence. Most recurrences appeared to be asymptomatic [7, 12].

Other causes for reoperation were failure of the fundoplication and persistent dysphagia. Dysphagia is a common side effect of the procedure, and an incidence of 13 % is reported in the literature [13]. This is in contrast with our findings in the quality of life questionnaires. Nineteen (23.8 %) patients in our study reported some kind of dysphagia after a median follow-up of 3 years. This difference can be explained by the fact that we operated on symptomatic patients with an ITS, whereas most studies reported results of patients with GERD symptoms. Another explanation for the high rate of persistent dysphagia might be due to a too tight wrap. Despite the high rate of dysphagia, the reported quality of life in this study was excellent and patients rated the overall procedure with a mean satisfaction of 8.4 on the VAS. This may be due to a reduction in severity of dysphagia rather than a complete relief of symptoms. It should be noted that this study described patients with an intrathoracic stomach and not with GERD. Therefore, these results may not be applicable to the population that receives a Nissen fundoplication for reflux disease, since this group of patients had other symptoms at baseline.

We found two major operative complications, which were a perforation of the esophagus during manipulation and a bleeding of the aorta leading to the death of the patient. To repair the perforation of the esophagus, an endoluminal stent was placed intraoperatively and the defect was closed with sutures. The leakage persisted due to partial necrosis of the esophageal wall. Therefore, a reoperation was necessary and eventually the patients had to undergo an esophageal resection and gastric tube reconstruction. An esophageal perforation or laceration is a feared yet known complication and has been reported previously in the literature. In all cases, this complication did not result in death of the patient [14, 15].

The second major complication was a perforation of the aorta. Aortic injuries during laparoscopic fundoplication are very rare and have only been detected twice in the past according to the literature [16, 17].

Another common complication of the Nissen fundoplication is a pneumothorax [18]. The reported incidence of this complication in the literature was 4 % in all Nissen fundoplications. However, the true percentage may be even higher since patients did not receive a routine X-thorax despite the fact that during surgery, the pleura is often opened. Five of our patients (6.25 %) developed a symptomatic pneumothorax, which received a thorax drainage in two cases. The reason for this increased incidence of pneumothoraces is that we only operated on patients with an ITS, whereas most literature describes only patients with GERD who are less prone to develop a pneumothorax.

In 20 of our cases, a mesh reinforced the cruroplasty. The choice for the use of a mesh application was made by individual preference of the operating surgeon. Although, the small sample size and unequal groups prohibit proper statistical comparison between patients that were treated with or without a mesh. Nevertheless, it is striking to see that no recurrences occurred in patients that received a mesh-reinforced cruroplasty. In addition, the complication rate and symptomatic outcome were comparable. Mesh related complications reported in the literature included erosion of the esophagus and migration or infection of the mesh [19]. We did not see any of these complications in our study. This can be explained by the fact that we reinforced the cruroplasty with a mesh only in the past few years. This is due to our increasing experiences with intrathoracic stomach repair and the presence of early recurrences in our cohort. As a result, we do not have any long-term follow-up data describing the mesh-based hiatal hernia repairs. A large meta-analysis published by Müller-Stich et al. in 2015 described the effect of mesh augmentation in hiatal hernia repairs in patients with a paraesophageal hernia. They concluded that the mesh reinforcement does reduce the recurrence rate without increasing the procedure related complications and mortality, at least for the mid-term follow-up. The reported mesh related complication rate in this meta-analysis was 1.7 % [20]. We used a synthetic mesh, but biological meshes are also available. However, the type of mesh that is more suitable for hiatal hernia repair has not yet been researched, and therefore, further randomized studies are needed.

## Conclusion

This large single center experience on laparoscopic repair of an intrathoracic stomach resulted in a high response rate. Although we do not have a preoperative comparison, fairly good results in patient satisfaction and symptom reduction were achieved after a median follow-up of 2.7 years. The symptomatic recurrence rate was very low, especially in the mesh-based cruroplasty.
